# Accuracy of Dental Photography: Professional vs. Smartphone's Camera

**DOI:** 10.1155/2021/3910291

**Published:** 2021-12-15

**Authors:** Carol Moussa, Louis Hardan, Cynthia Kassis, Rim Bourgi, Walter Devoto, Gilbert Jorquera, Saurav Panda, Roy Abou Fadel, Carlos Enrique Cuevas-Suárez, Monika Lukomska-Szymanska

**Affiliations:** ^1^Department of Restorative Dentistry, School of Dentistry, Saint-Joseph University, Beirut 1107 2180, Lebanon; ^2^Clinical Lecturer, Private and Referral Practice, Sestri Levante, Italy; ^3^Department of Prosthodontics, Universidad de los Andes, Santiago, Chile; ^4^Department of Periodontics and Oral Implantology, Institute of Dental Sciences, Siksha O Anusandhan University, 751002, India; ^5^Department of Periodontology, School of Dentistry, Saint-Joseph University, Beirut 1107 2180, Lebanon; ^6^Dental Materials Laboratory, Academic Area of Dentistry, Autonomous University of Hidalgo State, Circuito Ex Hacienda La Concepción S/N, San Agustín Tlaxiaca 42160, Mexico; ^7^Department of General Dentistry, Medical University of Lodz, 251 Pomorska St., 92-213 Lodz, Poland

## Abstract

There is a scant literature on the accuracy of dental photographs captured by Digital Single-Lens Reflex (DSLR) and smartphone cameras. The aim was to compare linear measurements of plaster models photographed with DSLR and smartphone's camera with digital models. Thirty maxillary casts were prepared. Vertical and horizontal reference lines were marked on each tooth, with exception to molars. Then, models were scanned with the TRIOS 3 Basic intraoral dental scanner (control). Six photographs were captured for each model: one using DSLR camera (Canon EOS 700D) and five with smartphone (iPhone X) (distance range 16-32 cm). Teeth heights and widths were measured on scans and photographs. The following conclusions could be drawn: (1) the measurements of teeth by means of DSLR and smartphone cameras (at distances of at least 24 cm) and scan did not differ. (2) The measurements of anterior teeth by means of DSLR and smartphone cameras (at all distances tested) and scan exhibited no difference. For documentational purposes, the distortion is negligeable, and both camera devices can be applied. Dentists can rely on DSLR and smartphone cameras (at distances of at least 24 cm) for smile designs providing comparable and reliable linear measurements.

## 1. Introduction

Nowadays, photography is playing a major part in the medical field, specifically in documentation [1–8]. The importance of digital photography has been brought to light during the COVID-19 pandemic where all interpersonal interactions relied mainly on online communication. Remote online medical consultations began to show a growing potential, and Telehealth became crucial for delivering virtual medical and educational support in all fields [9].

The use of photographs in dentistry has offered an updated perception of daily clinical practice. Apart from educational purposes, photography can be employed in treatment planning, tracking the evolution of the treatment, documenting, evaluating, communicating, publishing, lecturing, and marketing, artistic pictures, insurance, or legal purposes [10–13]. The Digital Smile Design (DSD) process is a methodical procedure relying on photographs and software analysis to determine esthetic outcomes; it has been used in the conception of esthetically pleasing smiles, from restorative dentistry to orthodontic treatment plans [14, 15]. Photographs are being captured by either a DSLR or a smartphone camera. Acquired photographic information has been described as an “objective and efficient communication tool among dentist, patient, and technician,” that can be used for smile design and mock-up techniques [16, 17].

For any clinician, practicality comes first and foremost, especially for frequently repeated procedures. In terms of feasibility, mobile dental photography (MDP) seems to be favorable in comparison with DSLR photography on account of smartphones' cost-effectiveness, considerably lighter weight, and faster learning curve [18]. The rate of dentists relying on the use of their smartphones' cameras, instead of professional DSLR cameras, is increasing exponentially because of the easy access and manipulation of the former [19].

DSLR cameras have specific settings and characteristics that dictate the protocols of capturing a photograph since it allows the photographer to control and change features such as aperture, exposure time, and international organization of standardization (ISO) sensitivity [18]. On their end, smartphone cameras perform automatic adjustments allowing the user to take a picture no matter the circumstances which can be both beneficial and disadvantageous at the same time. On the one hand, mobile phone facilitates the process of taking a picture [20], but on the other hand, if the user does not know how to properly manipulate the camera, the photograph can be captured in conditions that compel image distortion [21]. In fact, barrel effect is one of the problems that dentists face: it happens when the camera is too close to the subject and results in distorted image proportions. To eliminate this problem, the camera should be placed further away from the object; henceforth, the correct handling of the smartphone camera is essential [18].

With the growing importance of photography in the dental field, it seemed interesting to assess its accuracy and hence determine to what extent it is reliable for both DSLR and smartphone cameras. Therefore, the purpose of this study was to compare linear measurements of plaster models photographed with DSLR camera and smartphone's camera with linear measurements of digital models obtained with the intraoral dental scanner.

Two null hypotheses were tested: (1) there is no statistically significant difference among the linear measurements of teeth from digital images of the plaster model obtained from a DSLR, a smartphone's camera, or an intraoral dental scanner, and (2) the distance of the smartphone's camera to the object does not significantly affect the linear measurements in a photograph.

## 2. Material and Methods

### 2.1. Material

A total of thirty patients aged between 18 and 30 years with preserved natural dentition in maxillary teeth were selected for this study. Patients with gingival recessions, orthodontic anomalies, or prosthetic restorations in maxillary teeth, were excluded from this study. All signed a consent form accepting that their records can be used for educational purposes and studies. The study protocol was approved by the Institutional Review Board of Saint-Joseph University (FMD-202; ref.# USJ-2019-234).

### 2.2. Study Design

Dimensions recorded with the intraoral dental scanner were considered standard references because of the intraoral scanner's relatively determined accuracy [22–24]. Linear measurement's distortion was evaluated according to tooth placement, by comparing the width and height measurements of different teeth (central, lateral, canine, first, and second premolars) in photographs taken with the different devices. Moreover, this study compared the measurements in photographs captured at different distances between the smartphone camera and the plaster model.

Linear measurements of digital images of teeth from plaster models were evaluated according to the following factors: (1) device used: a DSLR (Canon EOS 700D with 100 mm macro lens) and a smartphone's camera (iPhone X); and (2) distance between the smartphone's camera and the object: 16 cm, 20 cm, 24 cm, 28 cm, and 32 cm. The sample size for each test had a power of at least 0.8 at a significance level of 0.05.

#### 2.2.1. Traditional Model Preparation

Thirty maxillary impressions were taken using standard trays and alginate impression material (Tropicalgin, Zhermak, Germany). The impressions were then immediately casted with type III dental stone (Elite Ortho, Zhermak, Germany). After setting, two reference lines (vertical and horizontal) were marked on each tooth from the right second premolar to the left second premolar, using a 0.3 mm pencil. The vertical lines were drawn from the zenith point perpendicularly to the middle of the incisal edge of the incisors or to the tip of the canine and premolar teeth. The horizontal lines were marked at the height of contour of each tooth.

**Figure 1:**
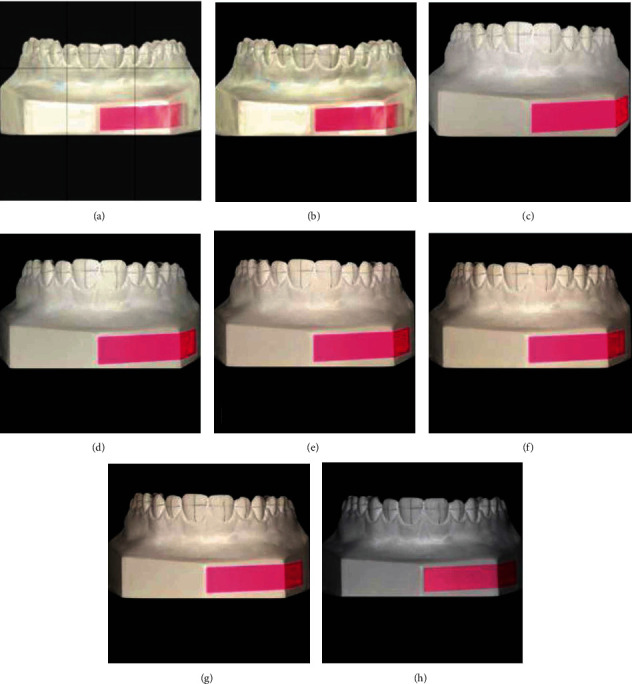
Photographs of the same model with the different camera devices. (a) Scan with grid lines to determine the positioning of the model. (b) Scan of the plaster model (dcm format to see the marked reference lines). (c) Photograph captured with iPhone X at 16 cm. (d) Photograph captured with iPhone X at 20 cm. (e) Photograph captured with iPhone X at 24 cm. (f) Photograph captured with iPhone X at 28 cm. (g) Photograph captured with iPhone X at 32 cm. (h) Photograph captured with Canon EOS 700D (100 mm macrolens).

To compute dimensions on a given scale, one fixed dimension should be included in all plaster casts' photographs. The known width (0.8 cm) of a prefabricated rectangular sticker was used as the fixed reference dimension for scale computation. A sticker was placed on the center of each cast's base in frontal view (Figure 1).

#### 2.2.2. Scanning Procedure

The models were first scanned with the 3Shape TRIOS scanner (TRIOS 3 Basic, 3Shape, Copenhagen, Denmark) for the control group, following the manufacturer's scan strategy protocol [25]. The three-dimensional (3D) scan of the cast had to be transferred into a two-dimensional (2D) representation so that linear measurements could be compared between the scan and the photographs. To do so, a frontal view of the scan had to be chosen so that it matches the frontal view depicted on the photographs. A see-through 2D square with gridlines dividing the square into thirds was superimposed on the scan, and the frontal view was chosen when the edges of the scanned model fit in the square and the base of the model coincided with the lower horizontal line of the grid.

#### 2.2.3. DSLR Photographs

DSLR cameras were positioned on a tripod while the plaster cast models were placed on a fixed stand. Six photographs were captured for each model: one photograph with the DSLR camera (Canon EOS 700D with 100 mm macrolens) and five photographs that were captured using a smartphone (iPhone X) whereby the settings were the same (Figure 1).

The picture size was chosen with a 1 : 1 aspect ratio for screen size (meaning that the width and height of the screen were equal, giving a square picture size), and grid lines dividing the screen into thirds were displayed on the screen. The lower horizontal line of the grid was superimposed over the base of the models. Each device's center of focus was directed to the incisor point, and the additional focal points were directed to the tips of the canines. All casts were captured by one operator to ensure standardization of the procedure.

The settings were fixed with a shutter speed of 1/125 and an aperture of F-22. The distance between the camera and the models was determined such that the cast's edges fit in the 1 : 1 camera frame, all while the cast was in focus.

#### 2.2.4. Smartphone Photographs

A feature of the iPhone X is that shutter speed and aperture size were automatically calibrated depending on the distance between the camera and the cast. The cast's edges had to also fit in the square camera frame of the phone. Five photographs were taken at different distances (determined after a pilot study): 16 cm, 20 cm, 24 cm, 28 cm, and 32 cm.

#### 2.2.5. Photograph Assessment

**Figure 2:**
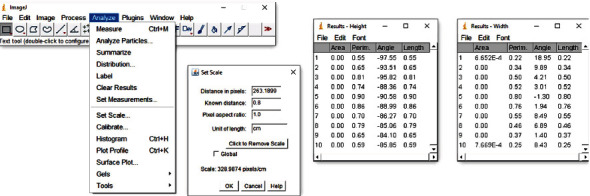
Example of setting a scale and measuring teeth heights and widths using ImageJ tools.

Afterwards, all photographs along with the chosen frontal view of each scanned model were assessed with the free software ImageJ (U.S. National Institutes of Health, Bethesda, Maryland, USA) with an accuracy of 0.01 mm. First, using the software, the width of the sticker (0.8 cm width) was marked on each photograph. The system measures this distance in pixels and sets a scale that automatically computes the measurements into the specified length unit (cm). Then, relying on the already drawn vertical and horizontal reference lines, teeth heights and widths were marked, and their measurements were recorded using the tool Measure Analyze. Measurements were taken in the same sequence for all the photographs, starting with the right second premolar and ending with the left second premolar (Figure 2).

### 2.3. Statistical Analysis

Data normality was verified using Shapiro–Wilk's test and the homoscedasticity using Levene's test. Statistical analyses were carried out according to the different experimental designs at a significance level of *α* = 0.05. The statistical tests were done using Sigma Plot 12.0 software. The width and height of each tooth were analyzed separately by means of a one-way ANOVA and Tukey test.

## 3. Results

The width of the first and second premolars significantly differed depending on the camera and the distance used (Table 1) (*p* < 0.001). For these teeth, values obtained from photographs captured with the smartphone camera at the distances of 24 cm, 28 cm, and 32 cm from the subject were statistically similar to those obtained with the DSLR camera. Moreover, values obtained from scans of premolars were statistically different from those relying on photos with mobile camera at the distance of 16 cm and 20 cm.

Comparison between teeth height in different photographs showed that there is no statistically significant difference (*p* > 0.05) (Table 2).

## 4. Discussion

The present study reported that the values measured using a DSLR and a smartphone camera recorded the same results for the frontal view of the scanned model. No statistically significant difference was found for neither width measurements (from canine to canine), nor height measurements (for all teeth) for both cameras. Additionally, there was no statistical difference between scans, DSLR, and smartphone cameras at distances greater than or equal to 24 cm. Thus, the first null hypothesis stating that there is no statistical difference between the two devices (DSLR and smartphone) can be partially rejected; it is true when the smartphone is at a distance of at least 24 cm from the object.

The only statistical difference between the two devices appeared for premolars' widths when the smartphone camera was at close distances of 16 cm and 24 cm. So, the second null hypothesis can be partially rejected since the distance of the smartphone's camera to the object affected the measured values of the first and second premolars' widths.

Photographs can help evaluate and assess smile esthetics while taking into consideration patient and clinician preferences [26, 27]. The treatment planning for esthetic cases relies mostly on frontal dental photographs where measures of teeth dimensions and proportions can be executed. Photograph analysis and processing techniques are increasingly being used for determining optimal thresholds of teeth shapes and dimensions along with soft tissue proportions [28–30]. The average widths of maxillary teeth in frontal view can be used to obtain esthetically pleasing smiles [31]. Moreover, digital imaging can be used for color measurements since it provides improved communication between the dentist and the laboratory technician [18]. The color matching ability of the observer showed a large variation, and photograph reliability in color matching was considered effective [32]. It was found that the application of photographs resulted in digital shade selection with a threshold within acceptable values. The used photographs can either be captured with a DSLR or a smartphone in adjunction to a suitable light since both devices gave similar reliable results [33]. The use of additional accessories for digital photography such as polarizing filters has also proved useful in shade matching [34]. These accessories are not exclusively used on DSLR cameras. Interestingly, the application of a crosspolarizing filter on a smartphone camera results in a more color-standardized photograph [35].

The digital approach in smile design has increasingly developed in the past years, given the growing role played by technology in daily life. DSD has become an interesting tool in esthetic dentistry. Computer and software resources are facilitating treatment planning and end-result predictions [36]. Many smile design systems already exist, like DSD, Cara Smile, Rebel Simplicity, Planmeca Romexis Smile Design, Aesthetic Digital Smile Design, Smile Designer Pro, and VisagiSMile [36–39]. Each smile design system has its own approach to analyzing a patient's smile and elaborate a treatment plan for an esthetically pleasing result [17, 39–41]].

Smartphone cameras are frequently being used by dentists since numerous studies focused on smartphone photographs and videos. This could be due to the fact that smartphone cameras are more practical giving their accessibility where a smartphone is a cheaper, lighter, and easier alternative for a DSLR camera, and it possesses the ability to record high quality photographs and videos [18]. Both tested cameras were chosen due to their availability, popularity, and quality of images. Moreover, Canon EOS 700D was chosen because it was used in other in vivo studies for teeth dimension measurements.

The current study compared the accuracy of 2D photographs between different devices. The results helped in evaluating the reliability of the photographs depending on the used device and its distance to the subject. It is known that when a camera device gets closer to a subject, more distortion occurs, especially on the picture's borders [42]. In this study, the 100 mm macrolens was used with the Canon EOS 700D because a longer distance was needed to put the subject in focus; so, minimal distortion occurred. For the smartphone camera, and because it can manage to adjust its camera settings automatically, different distances have been tested.

From a documentational point of view, independent of the camera device or the distance to the teeth, all photographs proved to be efficient. They can all provide sufficient and reliable information. No significant statistical difference was found between the two recordings (DSLR and smartphone cameras), and the accuracy of both was determined to be satisfying for clinical application [43].

Regarding measurement accuracy, the results showed that for teeth height, no statistical difference was noticed: photographs captured with the DSLR camera and the smartphone camera at all distances gave teeth height measurements statistically similar to the values obtained with the intraoral scanner. These results were accurate for all teeth independent of their position on the dental arch.

For width measurements, the results were tooth-dependent. Concerning the teeth from canine to canine, photographs captured with the DSLR camera and the smartphone camera at all distances gave values statistically similar to those measured with the intraoral scanner. Both cameras can be used at any distance, giving reliable and accurate measurements that can be used for smile design.

However, this study proved that the case was different for width measurements of premolars. This can be explained by the fact that premolars are more distally located on the curved dental arch; so, more distortion can affect them since they are located close to the edge of the photograph. In fact, photographs captured with the DSLR camera and the smartphone camera at a distance of 24 cm, 28 cm, and 32 cm gave premolar width measurements statistically similar to each other, but differing from the values obtained with the intraoral scanner. When it comes to photographs taken with the smartphone camera at a distance of 20 cm, the difference becomes statistically significant and even more so at 16 cm.

Clinical studies evaluating the variables examined in this article are scant. Moreover, randomized controlled clinical trials must be conducted to provide better insight into the accuracy of digital photography (DSLR and smartphone cameras) in terms of precision and trueness. Little information exists regarding the use of smartphones for photography in the dental field. Additionally, the impact of different sensors (for DSLR and smartphone cameras) is a factor that can influence smile evaluation on frontal view photographs [44].

Only maxillary casts were included in this study since measurements of teeth dimensions on photographs are mainly used for smile designs which in its turn tend to focus on maxillary teeth. Nonetheless, teeth measurements can be used in orthodontic treatments; therefore, mandibular casts will also be included in future studies. The reliability of DSLR and smartphone cameras can be assessed for factors other than dimension accuracy, such as shade or color evaluation. A similar study can also be conducted using a different type of smartphone, or even another study comparing dimension accuracy for photographs of patients' faces. Furthermore, since smartphone videos are already being used to record lip dynamics, to produce a 2D smile frame, and even to take dental impressions, further studies should be conducted to evaluate the accuracy of these videos. More research should be directed towards testing other DSLR cameras or smartphone cameras since the continuous evolution of camera specifications and photography technologies can alter the findings of the study.

## 5. Conclusions

Within the limitations of the present study, the following conclusions can be drawn:

1. The measurements of teeth by means of DSLR and smartphone cameras (at distances of at least 24 cm) and scan did not differ
2. The measurements of anterior teeth by means of DSLR and smartphone cameras (at all distances tested) and scan exhibited no difference

For documentational purposes, the distortion is negligible and both camera devices can be applied in clinical scenario. Moreover, dentists can rely on DSLR and smartphone cameras (at distances of at least 24 cm) for smile designs providing comparable and reliable linear measurements.

## Figures and Tables

**Table 1:** Mean (SD) in cm of the width of teeth obtained from different photographs.

Teeth	Scan	DSLR	16 cm	20 cm	24 cm	28 cm	32 cm
15	0.25 (0.05)^a^	0.24 (0.06)^ab^	0.17 (0.06)^c^	0.20 (0.07)^bc^	0.22 (0.07)^ab^	0.23 (0.06)^ab^	0.23 (0.06)^ab^
14	0.29 (0.04)^a^	0.29 (0.06)^ab^	0.22 (0.06)^c^	0.25 (0.06)^bc^	0.26 (0.06)^ab^	0.28 (0.05)^ab^	0.28 (0.05)^ab^
24	0.29 (0.04)^a^	0.27 (0.05)^ab^	0.22 (0.05)^c^	0.24 (0.05)^bc^	0.25 (0.05)^ab^	0.27 (0.05)^ab^	0.27 (0.05)^ab^
25	0.26 (0.06)^a^	0.23 (0.07)^ab^	0.19 (0.06)^c^	0.20 (0.06)^bc^	0.21 (0.06)^ab^	0.23 (0.06)^ab^	0.24 (0.06)^ab^

SD: standard deviation. Dental teeth are in international nomenclature. For teeth 13, 12, 11, 21, 22, and 23, differences between photographs were not statistically significant (*p* > 0.05). For each row, different lowercase letters indicate statistically significant differences (*p* < 0.05).

**Table 2:** Mean (SD) in cm of the height of teeth obtained from different photographs.

Teeth	Scan	DSLR	16 cm	20 cm	24 cm	28 cm	32 cm
15	0.53 (0.09)^a^	0.54 (0.09)^a^	0.50 (0.07)^a^	0.52 (0.08)^a^	0.52 (0.08)^a^	0.53 (0.08)^a^	0.53 (0.08)^a^
14	0.60 (0.07)^a^	0.61 (0.07)^a^	0.58 (0.08)^a^	0.59 (0.07)^a^	0.60 (0.07)^a^	0.60 (0.07)^a^	0.61 (0.07)^a^
24	0.62 (0.08)^a^	0.62 (0.07)^a^	0.59 (0.08)^a^	0.61 (0.08)^a^	0.61 (0.08)^a^	0.62 (0.08)^a^	0.63 (0.08)^a^
25	0.56 (0.07)^a^	0.56 (0.07)^a^	0.53 (0.06)^a^	0.55 (0.06)^a^	0.55 (0.07)^a^	0.55 (0.07)^a^	0.56 (0.07)^a^

SD: standard deviation. Dental teeth are in international nomenclature. For teeth 13, 12, 11, 21, 22, and 23, differences between photographs were not statistically significant (*p* > 0.05). For each row, different lowercase letters indicate statistically significant differences (*p* < 0.05).

## Data Availability

All data are included in the manuscript. If needed, authors can provide all study documentation.
